# Development of an MRI based artificial intelligence model for the identification of underlying atrial fibrillation after ischemic stroke: a multicenter proof-of-concept analysis

**DOI:** 10.1016/j.eclinm.2025.103118

**Published:** 2025-02-17

**Authors:** Zijie Zhang, Yang Ding, Kaibin Lin, Wenli Ban, Luyue Ding, Yudong Sun, Chuanliang Fu, Yihang Ren, Can Han, Xue Zhang, Xiaoer Wei, Shundong Hu, Yuwu Zhao, Li Cao, Jun Wang, Saman Nazarian, Ying Cao, Lan Zheng, Min Zhang, Jianliang Fu, Jingbo Li, Xiang Han, Dahong Qian, Dong Huang

**Affiliations:** a Heart Center, Shanghai Sixth People's Hospital Affiliated to Shanghai Jiao Tong University School of Medicine, Shanghai, China; b Department of Liver Surgery, Renji Hospital, Shanghai Jiao Tong University School of Medicine, Shanghai, China; c School of Biomedical Engineering, Shanghai Jiao Tong University, Shanghai, China; d Department of Cardiology, Shanghai Institute of Cardiovascular Diseases, Zhongshan Hospital, Fudan University, Shanghai, China; e School of Medicine, Shanghai Jiao Tong University, Shanghai, China; f Department of Radiology, Shanghai Sixth People's Hospital Affiliated to Shanghai Jiao Tong University School of Medicine, Shanghai, China; g Department of Neurology, Shanghai Sixth People's Hospital Affiliated to Shanghai Jiao Tong University School of Medicine, Shanghai, China; h School of Computer and Computational Science, Hangzhou City University, Hangzhou, China; i Section for Cardiac Electrophysiology, Department of Medicine/Cardiology, University of Pennsylvania Perelman School of Medicine, Philadelphia, PA, USA; j Department of Neurology, Huashan Hospital, Fudan University, Shanghai, China; k Department of Neurology, Minhang Hospital, Fudan University, Shanghai, China; l Department of Neurology, Shanghai Tenth People's Hospital, Tongji University School of Medicine, Shanghai, China

**Keywords:** Atrial fibrillation, Ischemic stroke, Convolutional neural network, Radiomics

## Abstract

**Background:**

Atrial fibrillation (AF) represents a major risk factor of ischemic stroke recurrence with serious management implications. However, it often remains undiagnosed due to lack of standard or prolonged cardiac rhythm monitoring. We aim to create a novel end-to-end artificial intelligence (AI) model that uses MRI data to rapidly identify high AF risk in patients who suffer from an acute ischemic stroke.

**Methods:**

This study comprises an internal retrospective cohort and a prospective cohort from Shanghai sixth people's hospital to train and validate an MRI-based AI model. Between January 1, 2018 and December 31, 2021, 510 patients were retrospectively enrolled for algorithm development and performance was measured using fivefold cross-validation. Patients from this trial were registered with http://www.chictr.org.cn, ChiCTR2200056385. Between September 1, 2022 and July 31, 2023, 73 patients were prospectively enrolled for algorithm test. An external cohort of 175 patients from Huashan Hospital, Minhang Hospital, and Shanghai Tenth People's Hospital was also enrolled retrospectively for further model validation. A combined classifier leveraging pre-defined radiomics features and *de novo* features extracted by convolutional neural network (CNN) was proposed to identify underlying AF in acute ischemic stroke patients. Area under the curve (AUC), sensitivity, specificity, accuracy, positive predictive value, and negative predictive value were calculated for model evaluation.

**Findings:**

The top-performing combined classifier achieved an AUC of 0.94 (95% CI, 0.90–0.98) in the internal retrospective validation group, 0.85 (95% CI, 0.79–0.91) in the external validation group, and 0.87 (95% CI, 0.90–0.98) in the prospective test group. Based on subgroup analysis, the AI model performed well in female patients, patients with NIHSS > 4 or CHA_2_DS_2_-VASc ≤ 3, with the AUC of 0.91, 0.94, and 0.90, respectively. More importantly, our proposed model identified all the AF patients that were diagnosed with Holter monitoring during index stroke admission.

**Interpretation:**

Our work suggested a potential association between brain ischemic lesion pattern on MR images and underlying AF. Furthermore, with additional validation, the AI model we developed may serve as a rapid screening tool for AF in clinical practice of stroke units.

**Funding:**

This work was supported by grants from the 10.13039/501100001809National Natural Science Foundation of China (NSFC, Grant Number: 81871102 and 82172068); 10.13039/501100008233Shanghai Jiao Tong University School of Medicine, Two-Hundred Talent Program as Research Doctor (Grant Number: SBR202204); Municipal Science and Technology Commission Medical Innovation Project of Shanghai, (Grant/Award Number: 20Y11910200); 10.13039/501100010167Research Physician Program of Shanghai Shen Kang Hospital Development Center (Grant Number: SHD2022CRD039) to Dr. Dong Huang and the SJTU Trans-med Awards Research (No. 20220101) to Dahong Qian.

**Research in context**

**Evidence before this study**

AF increases the risk of ischemic stroke but is often undetected. AI has shown promise in multiple clinical tasks, yet few studies have linked DWI stroke patterns to AF using advanced AI models. Existing research is limited by small sample sizes or retrospective designs, highlighting the need for a robust and interpretable AI tool with multicenter or prospective validations.

**Added value of this study**

We developed an AI model using radiomics and CNN features, trained on ischemic stroke cohorts from 4 hospitals. It achieved favorable performance in the internal retrospective testing group, external retrospective validation group and internal prospective group (AUC: 0.94, 0.85, 0.87, respectively) and provided interpretable insights through visualization algorithms. Furthermore, subgroup analysis suggested higher benefits in females and specific clinical populations (NIHSS > 4, CHA₂DS₂-VASc ≤ 3).

**Implications of all the available evidence**

The AI model we developed may serve as a rapid screening tool for AF in clinical practice of stroke units, so that prolonged cardiac rhythm monitoring and potential change of secondary prevention strategy could be administered in selected patients.

## Introduction

Ischemic stroke is the leading cause of death and disability worldwide that imposes a substantial economic burden.^1^ Thorough diagnostic evaluation to identify the underlying pathophysiology is warranted because the etiology facilitates treatment decision making. Atrial fibrillation (AF) is a well-recognized cause and accounts for up to one-third of ischemic strokes.^2^ AF-associated strokes are more often fatal and debilitating in comparison with strokes from other etiologies.^3^^,^^4^ However, due to the often transient and asymptomatic nature of AF, many patients remain unaware until ischemic stroke becomes the first manifestation.^5^^,^^6^ Aside from this causal relationship between AF and stroke, alternative hypotheses also exist. In one, strokes in certain brain regions (e.g., insular or brainstem) could cause cardiovascular disturbance by affecting autonomic pathways, resulting in neurogenically induced AF.^7^ In a second hypothesis, AF may be just a comorbidity to stroke since they share similar risk factors including advanced age and hypertension. Although it's still unclear if AF captured after stroke is the cause, an innocent bystander, or the consequence of stroke, the detection of AF represents a significant risk factor of stroke recurrence and may lead to the shift of secondary prevention strategy from antiplatelet medication to anticoagulation therapy.^8^ Thus, searching for underlying AF after ischemic stroke is paramount with serious management implications.

Currently, the standard procedure for post-stroke AF detection involves a resting ECG, followed by Holter monitoring.^9^ If etiology is still undetermined after sufficient diagnostic assessment, also referred to as embolic stroke of undetermined source (ESUS), “long-term rhythm monitoring is recommended” according to the 2021 American Heart Association (AHA)/American Stroke Association (ASA) guidelines.^10^ Nevertheless, firm guidance regarding the timing, method or duration of cardiac monitoring is not provided, unavoidably giving rise to significant heterogeneity in clinical practice across the world. In fact, it is reported that even 24 h Holter monitoring is routinely completed in a limited number of stroke centers owing to physician discretion or resource limitation.^11^^,^^12^ Therefore, a notable proportion of AF is underdiagnosed during the index stroke admission, calling for novel rapid and accurate AF screening modalities.

Diffusion-weighted imaging (DWI) remains the most sensitive magnetic resonance imaging (MRI) sequence for the detection of ischemic infarct core by measuring the Brownian motion of water molecules within tissue voxels.^13^^,^^14^ It is frequently performed in clinical settings as an integral part of the diagnostic workup for acute ischemic strokes. Previous studies have explored whether DWI lesion patterns could be used for the differential diagnosis of stroke etiologies. However, controversy over the parameter selection continues and the classification accuracy is far from satisfactory.^15^^,^^16^ Since artificial intelligence (AI) has shown advantages over traditional methodologies in numerous clinical tasks,^17^ we postulate that AI-assisted DWI images could be utilized to efficiently identify underlying AF. Our previous work^18^ has demonstrated the feasibility of an AI algorithm based on the combination of pre-defined radiomics features and *de novo* features extracted by convolutional neural network (CNN). In this study, the algorithm is trained in a larger internal retrospective cohort, validated in an external retrospective cohort and a separate prospective cohort, aiming to verify the concept that AF detection in ischemic stroke patients can be achieved through AI-augmented MRI images with notable accuracy and minimal labor cost.

## Methods

### Study design and patients

This study comprises an internal retrospective cohort for the establishment of the AI model and a separate prospective cohort on which the model performance was evaluated. The clinical trial was registered with http://www.chictr.org.cn, ChiCTR2200056385.

### Ethics

The study protocol was approved by institutional review board and the Ethics Committee of Shanghai Sixth People's hospital (Approval number: 2022-KY-032(K)). Written informed consent for de-identified clinical data usage in this research was obtained from each participant. Moreover, an external validation cohort from Huashan Hospital, Minhang Hospital, and Shanghai Tenth People's Hospital was enrolled to confirm the accuracy and reproducibility of the model. It was approved by the participating sites' institutional review boards and ethics committees (Approval number: SHSY-IEC-5.0/24K218/P01 from Shanghai Tenth People's Hospital, HIRB-2023621 from Huashan Hospital, 2023-022-01 K from Minhang Hospital), requirement for patient informed consent was waived due to its retrospective nature.

### Data collection

In the retrospective cohorts, patients were eligible for enrollment if they were diagnosed of acute ischemic stroke according to the AHA/ASA guidelines^10^ after index admission between January 2018 and December 2021 in Shanghai Sixth People's Hospital, or between January 2024 and June 2024 in Huashan Hospital, Minhang Hospital, and Shanghai Tenth People's Hospital. Acute ischemic stroke is defined as sudden neurologic dysfunction caused by focal brain ischemia with imaging evidence of acute infarction. Patients with symptoms lasting over 2 weeks are not considered acute. All the MRI were performed within 24 h after patients' index admission. Patients were allocated to definite AF group if they had: (i) medical history of known AF or (ii) newly diagnosed AF based on a workup consisting of 12-lead electrocardiogram, Holter monitoring of at least 24 h and transthoracic echocardiography (TTE). Other patients were classified into non-AF group. Exclusion criteria included the following: (i) no DWI scan available or DWI without adequate quality; (ii) Transient ischemic attack (TIA) or no identifiable acute infarction lesion on DWI; (iii) patients who did not get any rhythm monitoring or received a Holter monitoring less than 24 h; (iv) valvular heart disease (according to the definition of valvular disease guideline of 2024^19^); and (v) incomplete medical history. All the consecutive patients were enrolled if selection criteria were met. Personally identifiable information (e.g., name, email address, or social security number) were not collected in this study.

Between September 2022 and July 2023, an independent cohort of consecutive participants diagnosed with acute ischemic stroke were prospectively enrolled from Shanghai Sixth People's Hospital. The inclusion and exclusion criteria of the prospective cohort were identical to that of the retrospective cohort. The DWI image files were forwarded to AI model developers (Y.D and C.H) by radiologists (X.W and S.H) and personnel from both sides were unaware of the AF status of individual patients. The comparison between the predicted AF status generated by AI models and actual diagnosis after the AF workup was made by an independent researcher (Z.Z).

### Imaging protocol

Imaging protocol of DWI images from Shanghai Sixth People's Hospital and other participating cites were summarized in Supplementary Table S1.

### Data preprocessing

Acute infarct lesions in DWI images were manually segmented with ITK-SNAP (www.itksnap.org) by a radiologist (S.D., with 8 years of experience). The results were checked and segmentations with suboptimal quality were corrected by a senior neuroradiologist (X.W., with 15 years of experience in interpreting brain MRI studies). More specifically, to generate a segmentation mask, the researchers first reviewed all sequences to identify the ischemic lesions. And the infarcted areas were segmented on a layer-by-layer basis until the full lesion volume was included and a three-dimensional lesion volume could be reconstructed. To reduce variance in terms of channel count and image dimensions, which could pose challenges during training, we standardized all images to a uniform size of [32, 256, 256], which is larger than the dimensions of all our data. For the resulting noninformation areas, we have uniformly filled them with zeros. To ensure comparability of signal intensities across distinct patients, a normalization strategy was implemented and image intensities were rescaled to a fixed range.

### Model structure, training, and testing

**Fig. 1:**
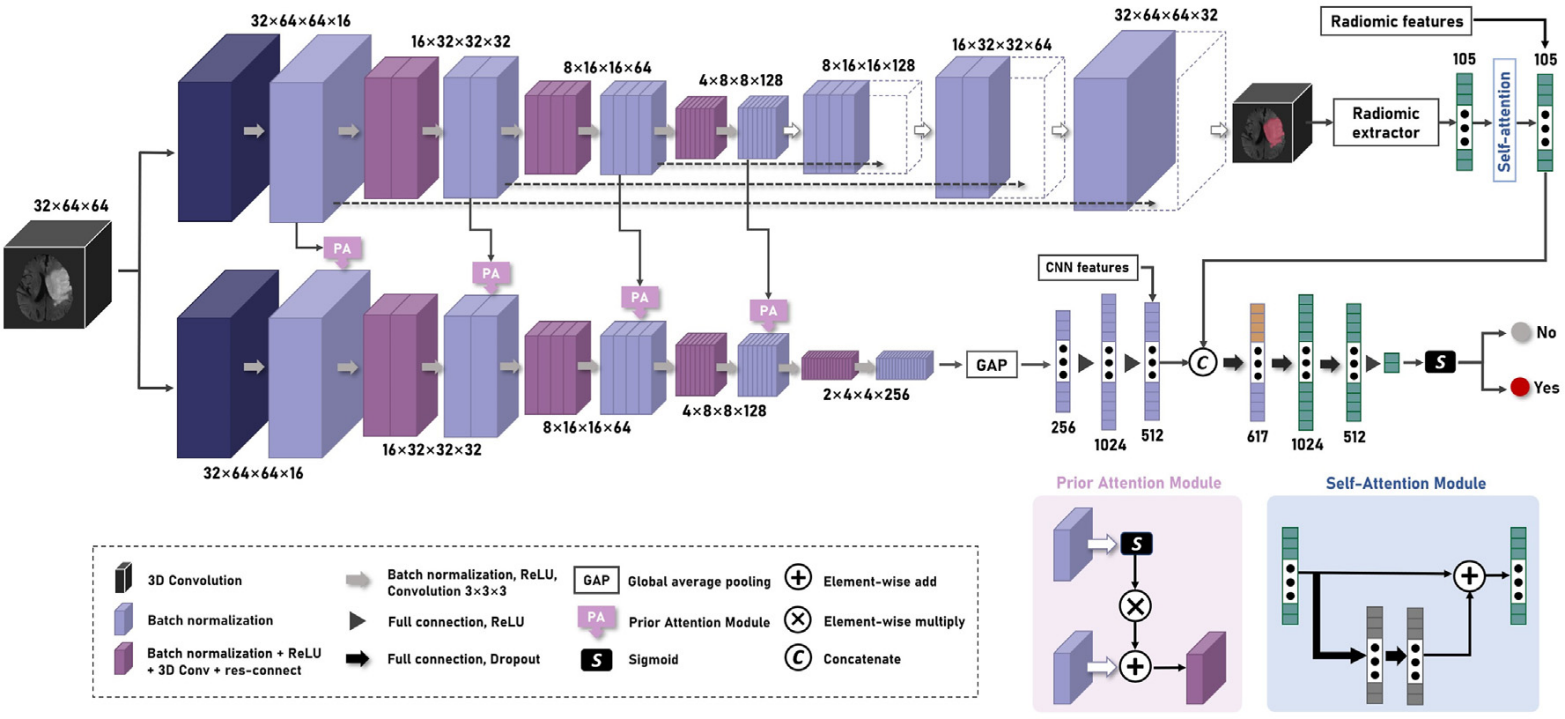
**The hierarchical architecture of the AI model**.

We implemented a multi-task framework designed for both lesion region segmentation and atrial fibrillation presence classification (Fig. 1). The two branches of the framework take the same multi-channel MRI images formatted to a size of 32 × 64 × 64 as input. The segmentation branch, utilizing a 3D U-Net structure, identifies lesion areas within the MRI images. Moreover, the prior attention guiding the classification branch is generated by the subsampling layers of the U-Net.

The classification branch consists of a 3D CNN network with convolution layers structured similarly to the subsampling layers of the U-Net, so that we can leverage the prior attention generated by the corresponding layers in segmentation branch to focus on the lesion part. In the final stage of segmentation branch, radiomic features are extracted from the lesion region, emphasizing relevant features through a self-attention module. The semantic features from the CNN are combined with radiomics features and passed through fully connected layers for ultimate classification.

For the segmentation loss function and classification loss function, we employed the Dice loss and sparse categorical cross-entropy, respectively. Three classification paths were considered: a radiomics-based classifier using only radiomics features, a CNN-based classifier using only CNN features, and a combined classifier employing both CNN and radiomics features. The 1D features underwent processing through a fully connected layer and a SoftMax activation layer, producing binary classification results for radiomics-based and CNN-based classifier paths. Using L_seg_ to denote segmentation loss, L_cnn_ for CNN-based classifier, L_rad_ for radiomics-based classifier, and L_com_ for combined classifier. Our multi-task framework operates in two stages. The first stage involves feature extraction, incorporating the CNN feature extractor, radiomics feature extractor, and the segmentation U-Net. More specifically, we utilized the opensource radiomics feature extraction library, pyradiomics, to extract features from the region of interest (ROI) (i.e., the thrombotic lesion area). The second stage, the classification stage, employs a multilayer perceptron (MLP) network for final classification using the combined features. The first stage utilizes the loss function (1 − L_seg_) + L_cnn_, and the second stage employs L_rad_ + L_com_ as loss function.

For training, an exponential decay learning rate (lr = lr_0_ e^−kt^) was employed, with a base learning rate (*lr*_*0*_) of 0.0001 and a decay rate (h) of 0.98. Other hyperparameters, derived from prior experience, included a total of 50 epochs, a batch size of 8, and the use of the Adam optimizer algorithm with exponential decay. Given the limited amount of data, to achieve a more robust model, we employed random rotations and flips of MRI images during the training process. This data augmentation technique helps increase the diversity of our training samples, allowing the model to learn more comprehensive features and reducing the risk of overfitting. Fivefold cross-validation was implemented by randomly assigning patients to five datasets. Within each fold, four datasets were utilized for training, and one for validation. The optimal model was selected based on the minimum validation loss observed throughout all training epochs. Following this, the generalization cohort underwent evaluation using the five models developed during the cross-validation of the primary analysis cohort. The predictions generated by these models were averaged to derive the overall performance.

### Performance evaluation

We assessed the classification effectiveness using metrics such as accuracy, sensitivity, specificity, positive predictive value, negative predictive value, and AUC. To bolster result reliability, we conducted a fivefold cross-validation on our internal dataset. The overall performance was evaluated by calculating the mean and standard deviation of the previously mentioned cross-validation metrics.

### Statistics

All statistical analysis was performed using IBM SPSS statistical software version 26 (IBM Corp., Armonk, NY, USA). Continuous variables were expressed as mean ± S.D. and their inter-group differences were examined by *t* test. Categorical variables are presented as frequency and percentage, and their inter-group differences were examined by χ^2^ test. Two-sided *P*-values less than 0.05 were considered indicative of a statistically significant difference in all tests.

### Role of funding source

The funder of the study had no role in study design, data collection, data analysis, data interpretation, or writing of the report.

## Results

### Patient characteristics

**Fig. 2:**
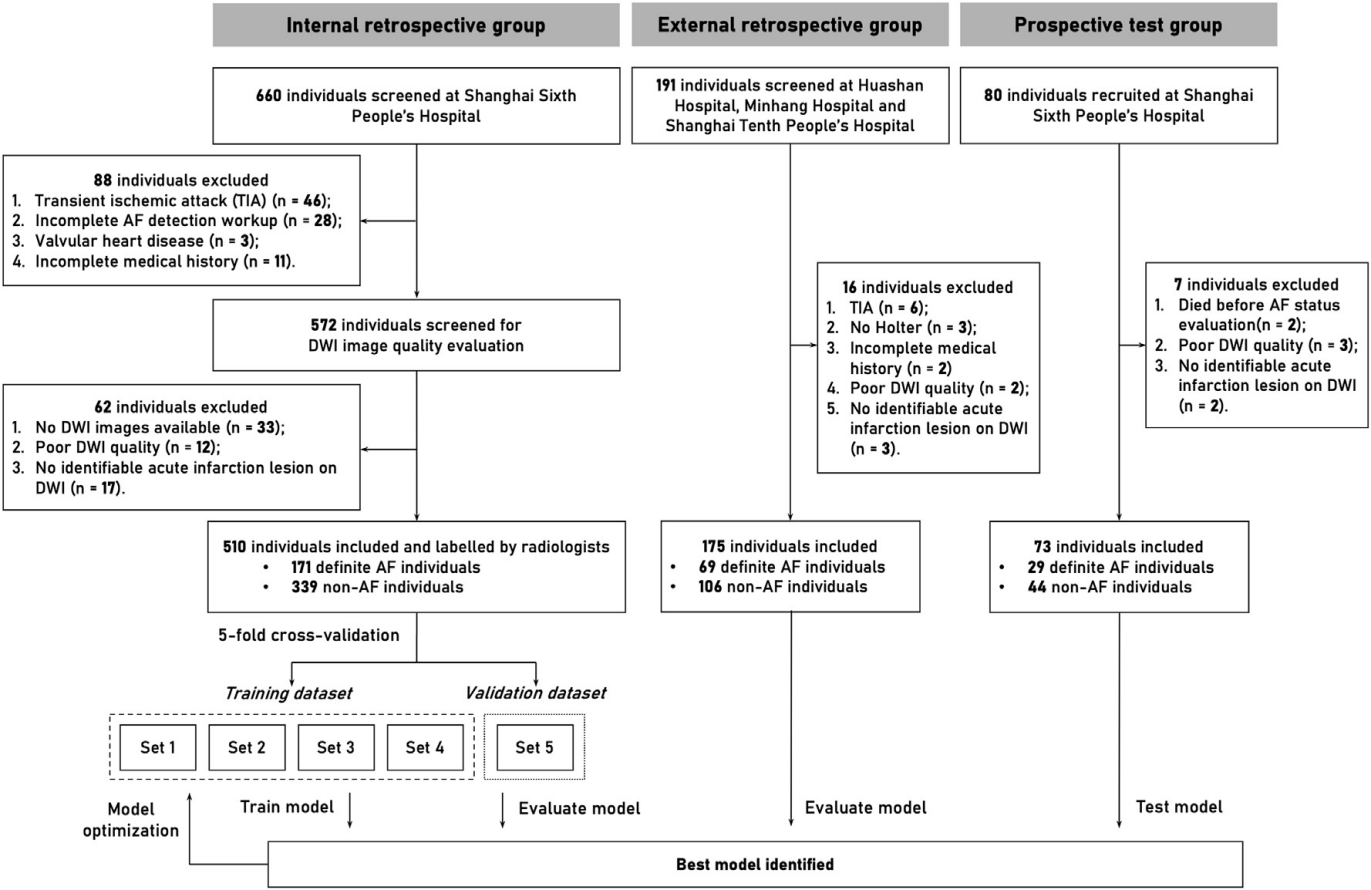
**Workflow diagram for patient enrollment as well as AI model development based on multicenter cohorts**.

Of the 660 patients available for eligibility screening in the internal retrospective group, 510 patients met the inclusion and exclusion criteria and were included for the AI model development. As to the external validation group, 191 patients were retrospectively reviewed and 175 were included. For the prospective test group, 80 patients were enrolled during the study period and the final analysis consisted of 73 patients. The study design and workflow are shown in Fig. 2. Table 1 indicates the demographic and clinical characteristics of the patients. The mean ± SD age of the recruited patients in three cohorts was 71.6 ± 12.0, 70.0 ± 11.1, and 71.8 ± 12.1, respectively. The mean ± SD score on NIH Stroke Scale (NIHSS) was 6.6 ± 6.9, 5.3 ± 6.1, and 7.2 ± 8.3. Among them, 171 (33.5%) patients in the internal retrospective group, 69 (39.4%) patients in the external validation group and 29 (39.7%) patients in the prospective test group had AF, either with a prior known AF history upon index admission or with newly detected AF during hospitalization. According to previous study,^20^ 92.7% of AF cases can be detected during a median of 64.0 h ECG monitoring. The average ECG recording duration was 67.6, 68.1, and 66.2 h in this study. Therefore, the underdiagnosis rate of AF should be relatively low. Also, compared to the internal retrospective group, patients in the external validation group and prospective test group had a lower CHA_2_DS_2_-VASc score (*P* < 0.05), which may be related to a higher proportion of paroxysmal AF. The incidence rate of comorbidities, including hypertension, diabetes mellitus, and coronary artery disease, were not statistically different between the internal retrospective group and prospective test group. However, the external validation group had significantly fewer patients with hypertension and diabetes (*P* < 0.001).

**Table 1:** Baseline characteristics of the study participants.

Characteristics	Internal retrospective group	External retrospective group	*P*^a^ value	Prospective group	*P*^b^ value
	(N = 510)	(N = 175)		(N = 73)	
Age -- yr	71.6 ± 12.0	70 ± 11.1	0.121	71.8 ± 12.1	0.894
Sex -- no. (%)			0.137		0.142
Male	333 (65.3)	125 (71.4)		54 (74.0)	
Female	177 (34.7)	50 (28.6)		19 (26.0)	
BMI -- kg/m^2^	24.8 ± 8.0	24.3 ± 3.2	0.421	23.4 ± 3.7	0.141
AF status -- no. (%)			0.158		0.297
Non-AF	339 (66.5)	106 (60.6)		44 (60.2)	
Definite AF	171 (33.5)	69 (39.4)		29 (39.7)	
CHA_2_DS_2_-VASc score			0.006		0.043
1	0	2 (2.9)		0	
2	3 (1.8)	6 (8.7)		2 (6.9)	
3	21 (12.3)	10 (14.5)		3 (10.3)	
4	39 (22.8)	21 (30.4)		12 (41.4)	
5	47 (27.5)	11 (15.9)		9 (31.0)	
6	46 (26.9)	11 (15.9)		3 (10.3)	
>6	15 (8.8)	8 (11.6)		0	
Paroxysmal AF	53 (31.0)	29 (42.0)	0.071	11 (37.9)	0.389
Newly detected AF	55 (32.1)	15 (21.7)	0.108	6 (20.7)	0.215
Average Holter duration -- h	67.6 ± 6.1	68.1 ± 5.9	0.346	66.2 ± 5.8	0.066
Score on NIH stroke scale	6.0 ± 6.9	5.3 ± 6.1	0.234	7.2 ± 8.3	0.177
Prior stroke or TIA -- no. (%)	115 (22.5)	22 (12.6)	0.004	15 (20.6)	0.701
Hypertension -- no. (%)	378 (74.1)	64 (36.6)	<0.001	55 (75.3)	0.823
Diabetes -- no. (%)	148 (29.0)	77 (44.0)	<0.001	25 (34.2)	0.361
Coronary artery disease -- no. (%)	53 (10.4)	12 (6.9)	0.169	7 (9.6)	0.833
Left atrial diameter >40 mm -- no. (%)	125 (24.5)	65 (37.1)	0.001	15 (20.6)	0.459

Data presented as mean ± standard deviation or n (%).

AF = atrial fibrillation; BMI = body mass index; TIA = transient ischemic attack.

a *P* value was obtained by comparison of the internal retrospective and external retrospective groups.

b *P* value was obtained by comparison of the internal retrospective and prospective groups.

### Model performance

According to the workflow diagram in Fig. 2, the five subsets of our retrospective dataset were used to develop five independent models. Each model was trained using four subsets and tested using the remaining subset. For each model, diagnostic performance of the radiomics-based classifier, the convolutional neural network (CNN)-based classifier, and the combined classifier based on the combination of radiomics features and semantic features extracted from CNN were evaluated, respectively. The statistical results tabulated in Table 2 indicate the overall performance of the five models. The combined classifier outperformed the other classifiers and achieved an average area under the curve (AUC) of 0.87 ± 0.06 in the retrospective validation group. Using the operating points with the maximum sum of sensitivity and specificity, the algorithm had an average sensitivity of 87.66% ± 13.35%, specificity of 79.35% ± 5.87%, and accuracy of 82.16% ± 2.35%. These results corresponded to an average positive predictive value (PPV) of 68.82% ± 4.31% and negative predictive value (NPV) of 93.57% ± 5.89%.

**Table 2:** Fivefold cross-validation results indicating diagnostic performances of different classifiers.

	AUC	Sensitivity	Specificity	Accuracy	PPV	NPV
Radiomics classifier	0.81 ± 0.06	85.33 ± 11.34	70.79 ± 4.18	75.69 ± 1.69	59.59 ± 1.3	91.28 ± 5.83
CNN classifier	0.69 ± 0.04	57.75 ± 14.29	78.70 ± 12.83	71.76 ± 4.70	61.06 ± 9.26	79.41 ± 4.02
Combined classifier	**0.87 ± 0.06**	**87.66 ± 13.35**	**79.35 ± 5.87**	**82.16 ± 2.35**	**68.82 ± 4.31**	**93.57 ± 5.89**

Except AUCs, data are percentages presented as means ± S.D. Performance is presented as AUC, sensitivity, specificity, accuracy, PPV, and NPV according to the optimal selected cutoff.

CNN = convolutional neural networks; AUC = area under the receiver operating characteristic curve; PPV = positive predictive value; NPV = negative predictive value.

**Fig. 3:**
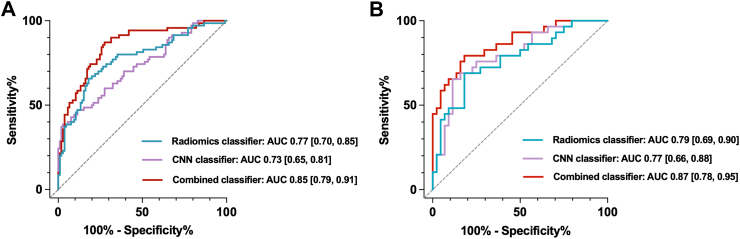
**Algorithm performance for detecting atrial fibrillation. (A)** ROC curve of the external validation group; **(B)** ROC curve of the prospective test group. AUCs of different classifiers are indicated respectively. ROC = receiver operating characteristic; AUC = area under the receiver operating characteristic curve.

Among the five models, Model 5 demonstrated the best performance in the retrospective cohort and was further tested in the external validation group and independent prospective group. The combined classifier in this model showed an AUC of 0.94 (95% CI, 0.90, 0.98) in the retrospective validation group, 0.85 (95% CI, 0.79–0.91) in the external validation group, and 0.87 (95% CI, 0.78, 0.95) in the prospective test group. The sensitivity, specificity, accuracy, PPV, and NPV for the internal validation group were 97.14% ± 3.23%, 80.60% ± 7.67%, 86.27% ± 6.68%, 72.34% ± 8.68%, and 98.18% ± 2.59%, respectively, those for the external validation group were 67.78% ± 6.92%, 89.41% ± 4.56%, 78.28% ± 6.11%, 87.14% ± 4.96%, and 72.38% ± 6.63%, respectively, whereas those for the prospective test group were 79.31% ± 7.86%, 89.04% ± 6.06%, 86.27% ± 6.68%, 74.19% ± 8.50%, and 91.55% ± 5.40%, respectively. Receiver operating characteristic (ROC) curves for atrial fibrillation prediction by different classifiers are shown in Fig. 3 and the detailed performance of radiomics classifier and CNN classifier in the test group are summarized in Table 3 and Table 4. The combined classifier exhibited the best overall performance in comparison with others.

**Table 3:** Diagnostic performances of different classifiers in external validation group.

	AUC	Sensitivity	Specificity	Accuracy	PPV	NPV
Radiomics classifier	0.77 [0.70, 0.85]	70.77 [64.03, 77.51]	78.18 [72.06, 84.3]	75.43 [69.05, 81.81]	65.71 [58.68, 72.75]	81.90 [76.2, 87.61]
CNN classifier	0.73 [0.65, 0.81]	**73.33** **[66.78, 79.89]**	71.54 [64.85, 78.22]	72.00 [65.35, 78.65]	47.14 [39.75, 54.54]	**88.57** **[83.86, 93.29]**
Combined classifier	**0.85** **[0.79, 0.91]**	67.78 [60.85, 74.70]	**89.41** **[84.85, 93.97]**	**78.28** **[72.18, 84.39]**	**87.14** **[82.18, 92.10]**	72.38 [65.76, 79.01]

Data in brackets are 95% confidence intervals.

CNN = convolutional neural networks; AUC = area under the receiver operating characteristic curve; PPV = positive predictive value; NPV = negative predictive value.

**Table 4:** Diagnostic performances of different classifiers in prospective test group.

	AUC	Sensitivity	Specificity	Accuracy	PPV	NPV
Radiomics classifier	0.79 [0.69, 0.90]	65.52 [56.29, 74.74]	**93.15** **[88.25, 98.05]**	85.29 [78.42, 92.17]	**79.17** **[71.29, 87.05]**	87.18 [80.69, 93.67]
CNN classifier	0.77 [0.66, 0.88]	62.07 [52.65, 71.49]	89.04 [82.98, 95.10]	81.37 [73.82, 88.93]	69.23 [60.27, 78.19]	85.53 [78.70, 92.35]
Combined classifier	**0.87** **[0.78, 0.95]**	**79.31** **[71.45, 87.17]**	89.04 [82.98, 95.10]	**86.27** **[79.60, 92.95]**	74.19 [65.70, 82.69]	**91.55** **[86.15, 96.95]**

Data in brackets are 95% confidence intervals.

CNN = convolutional neural networks; AUC = area under the receiver operating characteristic curve; PPV = positive predictive value; NPV = negative predictive value.

### Model interpretation and significant features for the prediction

**Fig. 4:**
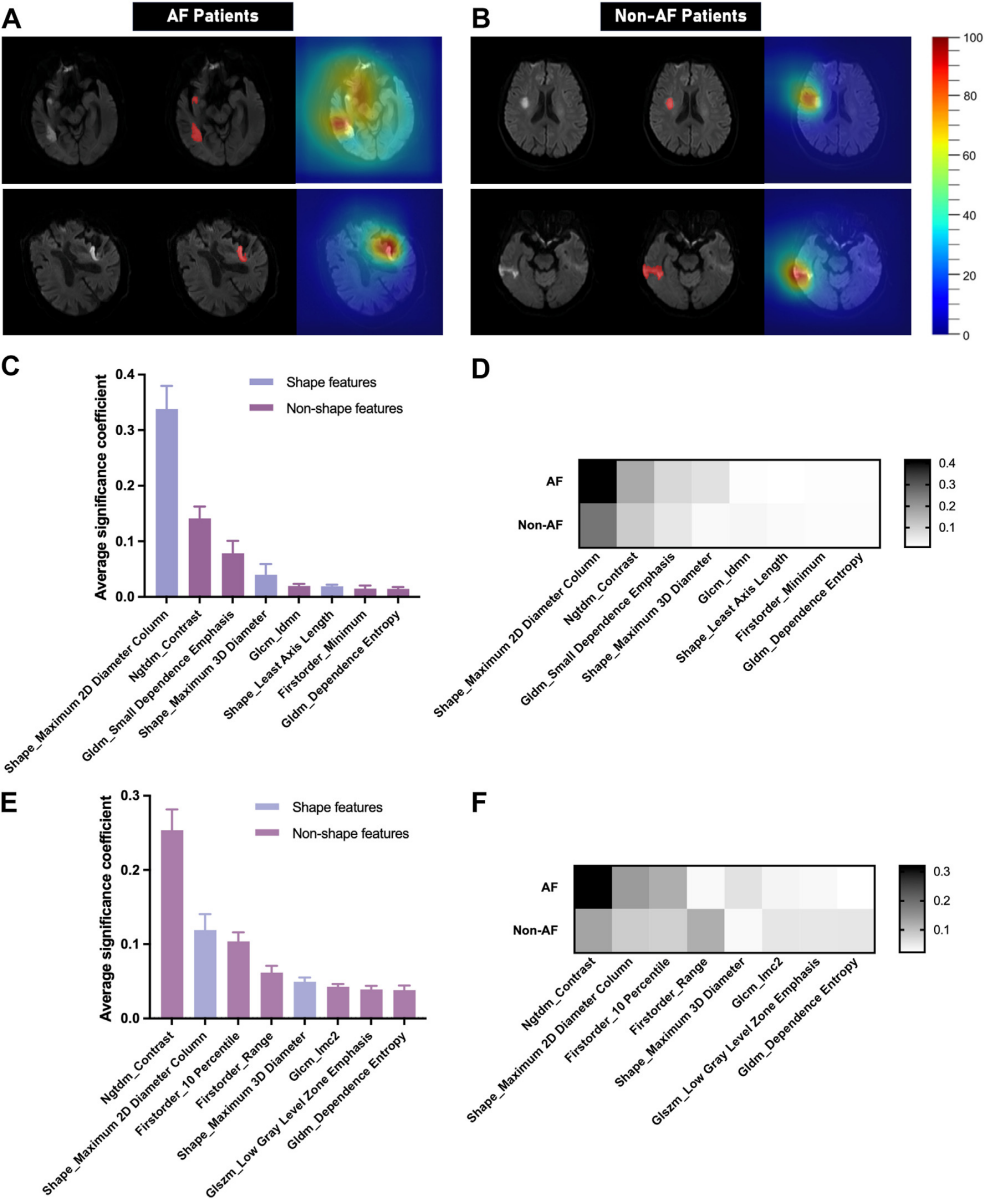
**Model visualization and statistical analysis of the feature significance for AF prediction. (A, B)** Convolutional feature map visualization to demonstrate the “attention mechanism” of our proposed AI model corresponding to four representative patient examples from AF and non-AF group (color masks the significant regions for the prediction, with the spectrum from blue-to-red associated with low-to-high significance); Also, we plotted the average significance of the top 8 radiomics features for AF prediction in the external validation group **(C)** and prospective test group **(E)**; Heatmap of the average significance of each radiomics feature in AF and Non-AF patient from the external validation group **(D)** and prospective test group **(F)** are shown, with the spectrum from black-to-white indicating strong-to-weak relevance with AF/non-AF classification.

To understand the regions of the MR images and the types of radiomics features that are highly related to the underlying atrial fibrillation prediction, we visualized convolutional feature maps using the Grad-CAM^21^ technique and calculated the average significance of each radiomics feature based on the feature significance ranking (FSR) mechanism.^22^ Fig. 4A and B shows feature maps of four representative patients and demonstrates that our proposed AI model mainly focused on the lesion regions of MR images to make decisions regarding AF and non-AF patients.

Then, we plotted the statistical significance of the top 8 radiomics features weighted by the self-attention module in Fig. 4C and E from the external validation group and prospective test group, respectively. Certain NGTDM feature, shape feature, GLCM feature, and GLDM feature are revealed to be important for the prediction due to their repeated presence in both groups and can be recognized as domain-invariant features. To further illustrate their contributions to the neural network, we created the heatmaps of the aforementioned radiomics features (Fig. 4D and F). The heatmap demonstrates that NGTDM feature and shape features are the top ones for the identification of AF patients, whereas the first order range feature, GLCM feature, GLSZM feature, and GLDM feature are the most important ones for the prediction of non-AF patients.

### Subgroup analysis of model performance

**Fig. 5:**
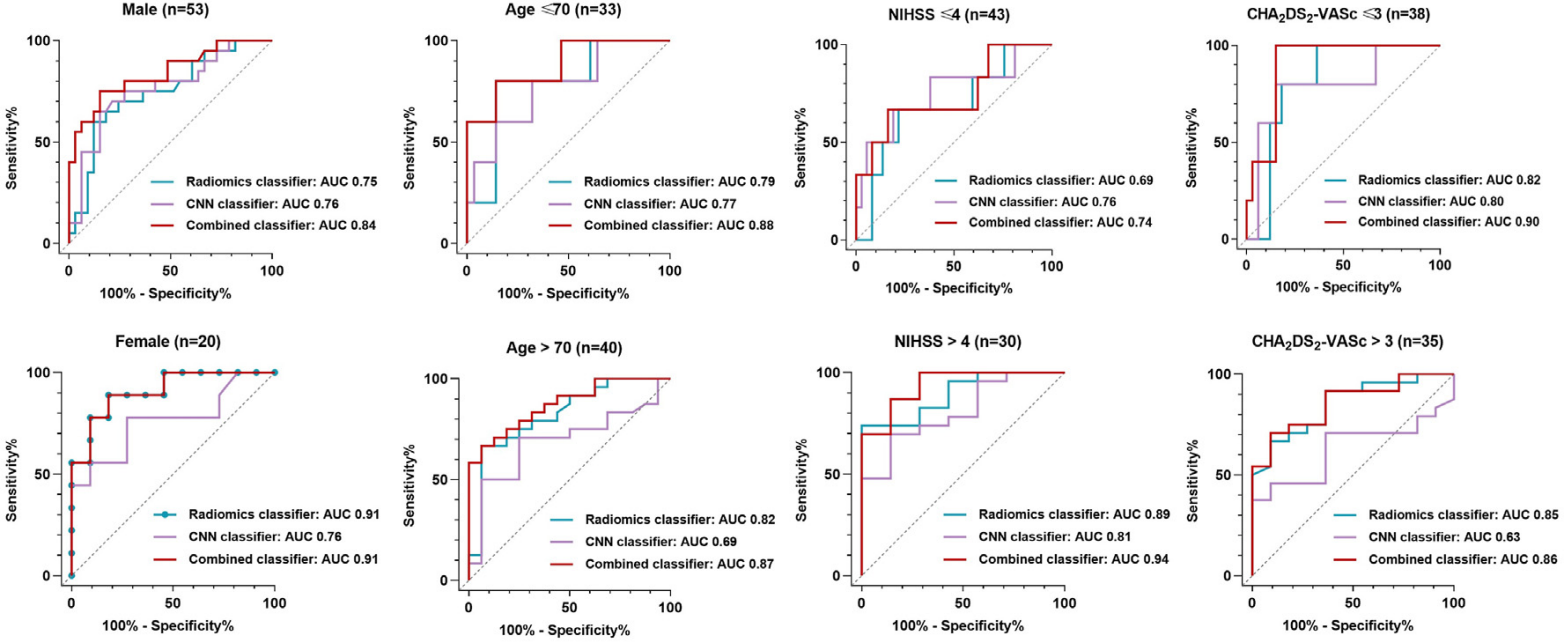
**Subgroup analysis of algorithm performance in the prospective cohort**. AUCs of different classifiers are indicated across specific subgroups defined by gender, age, NIHSS, and CHA_2_DS_2_-VASc scores. AUC = area under the receiver operating characteristic curve; NIHSS = NIH stroke scale.

Fig. 5 summarizes the algorithm performance in subgroups of the prospective test dataset. Apart from patients with NIHSS ≤ 4, the combined classifier had better performance in comparison with radiomics classifier and CNN classifier in other subgroups. And the combined classifier had similar performance in patients ≤70 years old and >70 years old, with AUC of 0.75 and 0.76, whereas a better performance in female patients, patients with NIHSS > 4 or CHA_2_DS_2_-VASc ≤ 3, with AUC of 0.91, 0.94, and 0.90, respectively.

**Fig. 6:**
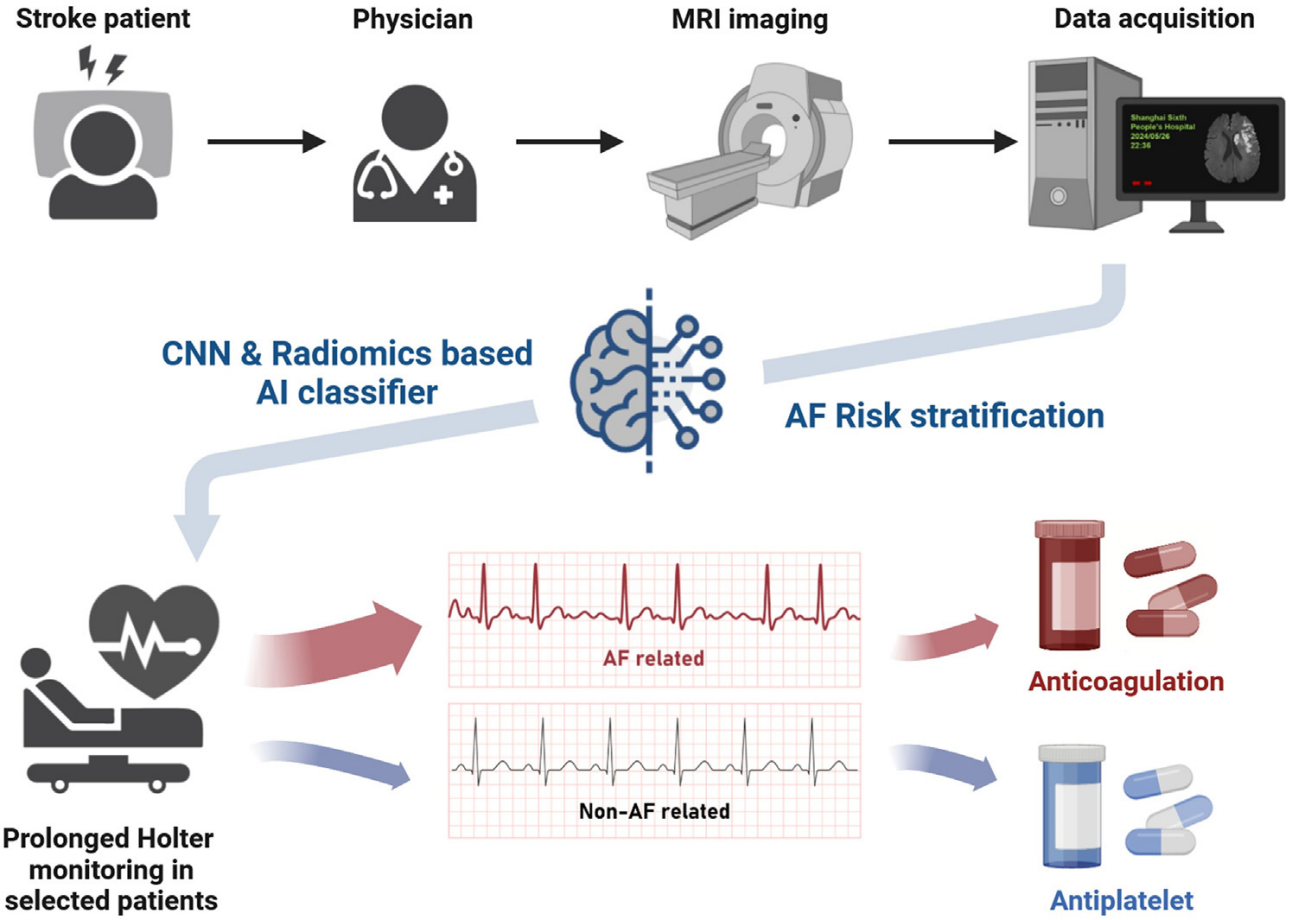
**Schematic of potential clinical application of the artificial intelligence model** (This figure was created with BioRender).

Moreover, since some patients were diagnosed with AF for the first time through long-term rhythm monitoring during admission, we further investigated the predictive ability of our proposed AI model in this subgroup. Surprisingly, the combined classifier had a high sensitivity of 100% and successfully identified all of the prior unknown paroxysmal AF or newly onset AF patients which were diagnosed during admission, in both retrospective validation group and prospective test group (Fig. 6).

## Discussion

In this study, we constructed and tested three different AI models: a radiomics classifier, a convolutional neural network (CNN) classifier, and a combined classifier to detect underlying AF in acute ischemic stroke patients based on DWI scan images. Our results suggested that the overall performance of radiomics approach or CNN approach alone was inferior to that of the combined approach. And the combined classifier exhibited satisfactory performance with an area under the curve (AUC) and accuracy of 0.94 and 86.27% in the internal retrospective group, 0.85 and 89.41% in the external validation group and 0.87 and 86.27% in the prospective test group.

To detect the majority of unknown paroxysmal AF or newly onset AF after stroke, current ESC guideline recommended a short-term ECG recording followed by continuous ECG monitoring for at least 72 h (class I, level B). However, the high costs and limited resources often hamper broad application in clinical settings. Thus, several studies have explored the AF risk stratification methods in ischemic stroke patients so that prolonged ECG monitoring could be applied in key population to increase the yield of investigation. Descriptors include demographics,^23^ obesity,^24^ simple clinical risk factors such as hypertension, heart failure history,^25^ and ECG (i.e., P-wave characteristics^26^), cardiac imaging (i.e., left atrial volume index^27^) or blood biomarkers (i.e., systemic immune inflammation index^28^). Nevertheless, these predictors are either nonspecific with limited odds ratio or intricate and involve multiple workups. In this study, we only included DWI scan images upon admission and our end-to-end AI algorithm represents a simple, cost-effective approach with high sensitivity and specificity, holding a promise of being incorporated into the clinical practice of rapid post-stroke AF screening.

To address the “black box” issue of the AI model, we embedded the FSR mechanism in the framework to extract significant radiomics features for the final predictions and employed class activation mapping (CAM) to visualize the attention mechanism of CNN branch. For the radiomics classifier, we noticed that most of the top 8 significant features are texture properties that are hard to quantify by human experts. For instance, topping that list was a neighboring gray tone difference matrix (NGTDM) feature, and it quantifies the difference between a gray value and the average gray value of its neighbors within a predefined distance. Such finding aligns with a previous post hoc analysis of the CRYSTAL AF study, in which the authors claim that the detection of a first episode of AF did not correlate with perceivable brain lesion pattern (i.e., shape or number).^29^ However, some of the radiomics features did have a histopathology basis behind them. For instance, Yip et al. found that NGTDM feature in PET/CT images was significantly associated with epidermal growth factor receptor mutation status of lung cancer.^30^ Moreover, Pyka et al. demonstrated that NGTDM feature enabled differentiation between tumor grades III and IV in glioma patients.^31^ Although this evidence originates from oncology research, it provides valuable insights for future efforts to explore the relationship between radiomics features, especially those domain-invariant ones, and microstructural changes in brain regions associated with AF, which could offer additional clinical rationale for the model's accuracy. For the CNN classifier, with the aid of feature heatmap, we found high signals inside ischemic lesion. Moreover, in patients with multiple lesions, the attention pattern is also multifocal. Collectively, these results highlight the reliability and scientific basis of our proposed AI model. In future studies, our pre-trained model, especially the automated segmentation branch, can be leveraged for the development of other MRI-based AI classifiers (e.g., identification of valvular heart disease related AF or atrial cardiomyopathy by cardiovascular MR imaging) by transfer learning approach.

Previous studies have demonstrated that the CHA_2_DS_2_-VASc scheme, although originally developed for another purpose, could also be potentially useful for identifying underlying AF in stroke patients. ECG monitoring in patients with high CHA_2_DS_2_-VASc score is likely to give high yield.^25^ However, for cases with relatively low CHA_2_DS_2_-VASc score, it remains to be difficult to determine their AF risk. Neurologists would be more intrigued in whether the model could precisely differentiate the AF status in this group. Therefore, we performed a subgroup analysis, and it revealed that the combined classifier performed well when the patients had CHA_2_DS_2_-VASc score ≤ 3, with an AUC of 0.90 in the prospective test group. And such result adds values to the future clinical translation of our proposed AI model.

There are two potential application scenarios for our algorithm. First, since the most appropriate clinical approach for selection of stroke patients to undergo more intensive cardiac monitoring has yet to be established, our algorithm can evaluate underlying AF probability during the initial MR imaging assessment upon admission to help guide further diagnostic tests, especially in female patients, patients with NIHSS > 4 or CHA_2_DS_2_-VASc ≤ 3. Considering that AF monitoring in stroke patients remains suboptimal in real-world clinical practice, the integration of this AI tool into picture archiving and communication system (PACS) and subsequent clinical workflows will serve as an effective complement to ECG and Holter monitoring, especially in resource-limited settings. Second, given the high sensitivity of our AI model in identifying prior unknown paroxysmal AF and newly onset AF after stroke, it holds promise for being an indicator of atrial cardiopathy in stroke patients and guides the administration of direct oral anticoagulant (DOAC). Prior trials, including NAVIGATE ESUS^32^ and RE-SPECT ESUS,^33^ were negative in demonstrating that DOAC therapy prevents stroke recurrences after ESUS. After that, researchers hypothesized that certain high-risk subgroups, for instance, patients with atrial cardiopathy, which has been linked to a greater risk of stroke, may ultimately benefit. However, the ARCADIA trial also failed to show the effectiveness of NOAC even though selected ESUS patients with evidence of atrial cardiopathy (ECG, echocardiography, and NT-proBNP) were enrolled.^34^ We postulate that these negative results may derived from the nonspecific selection criteria and our AI model could be useful as an adjunctive test.

Our study had some limitations. First, although the number of acute ischemic patients we enrolled in the retrospective cohort was adequate for AI model development and proof-of-concept, and a multi-center retrospective validation cohort was included to preliminarily confirm the accuracy and reproducibility of our model, the prospective cohort was a single-center study with limited patients which might produce bias to some extent. Future study including multicenter prospective cohorts with larger sample size can be used to test the extensibility of our proposed model. Second, the AF group was defined based on medical history, ECG or Holter monitoring upon admission, and the average duration of Holter recording in this study is 66–68 h, causing a certain degree of underdiagnosis inevitably. Third, comprehensive follow-up after discharge is necessary to identify more newly diagnosed AF patients. In fact, we did conduct a follow-up for the retrospective cohort in Jan 2024, trying to find out newly discovered AF cases after their discharging. However, due to the non-interventional nature of this trial, the rate of long-term rhythm monitoring after discharging, including insertable cardiac monitoring (ICM) and portable cardiac monitoring (e.g., smart watch), is only 18%. Among them, we did not find any newly onset AF cases. Moreover, 21% of the patients were dead at the time of our follow-up. According to our own data and previous studies, it is estimated that the classification accuracy of 3%–5% of the cohort may be affected due to undetected AF patients in ESUS population. While such an impact exists, it is not large enough to significantly change the overall conclusions of our study. We believe such limitation in this proof-of-concept study will be overcome in an interventional prospective trial in the future, so that patients who are later found to have AF after discharge, especially those who are classified into ESUS during admission, can be taken into consideration to train a more robust AI model.

Moreover, we observed some discrepancies between our datasets and previous literatures regarding the AF rate and proportion of female patients. The AF rates in our cohorts, ranging from 33.5% to 39.7%, were higher than expected. We postulate that the following factors might explain this discrepancy: (i) patients enrolled in this study were derived from tertiary care settings with a higher likelihood of treating complex cases, such as those with known or suspected AF; (ii) the average duration of cardiac monitoring in our cohort was 66–68 h, which increased the likelihood of AF detection; and (iii) the demographic characteristics or the epidemiology of stroke in the Shanghai region may also contribute to the difference. As to the lower proportion of female patients in our cohorts, we believe that it also reflects the regional or institutional variations rather than selective enrollment. Despite these discrepancies, the reliability and robustness of our AI model might not be affected since we employed an end-to-end training approach using only MR images as input without incorporating demographic information such as gender. Additionally, the model was designed to capture subtle imaging markers associated with underlying AF, irrespective of its prevalence in the dataset. Therefore, our imaging-driven approach ensures that the model's performance is generalizable across different patient populations.

In conclusion, our study proposed an advanced MRI-based artificial intelligence model for the rapid detection of underlying AF in post-stroke patients. The model leverages self-attention mechanism to extract both radiomics features and CNN features to produce favorable prediction accuracy, providing clinically beneficial information for treatment decision making. Future multi-national prospective studies are needed to further optimize this model for broader clinical application.

## Contributors

Concept and design: Zijie Zhang, Yang Ding, Kaibin Lin, Jingbo Li, Dahong Qian, Dong Huang. Acquisition, analysis, or interpretation of data: Wenli Ban, Luyue Ding, Yihang Ren, Can Han, Xue Zhang, Xiaoer Wei, Yudong Sun, Yuwu Zhao, Li Cao, Jun Wang, Ying Cao, Lan Zheng, Min Zhang, Jianliang Fu. Drafting of the manuscript: Zijie Zhang, Yang Ding. Critical review of the manuscript for important intellectual content: Saman Nazarian. Statistical analysis: Zijie Zhang, Yang Ding, Kaibin Lin. Supervision: Xiang Han, Jingbo Li, Dahong Qian, Dong Huang. Zijie Zhang and Dong Huang directly accessed and verified the underlying data. All authors have read and approve the final version of the manuscript.

## Data sharing statement

Data generated or analyzed during the study are available from the corresponding author by request.

## Declaration of interests

Dr. Saman Nazarian reports grants from US NIH NHLBI, grants from ADAS Software, grants from Biosense Webster and consulting fee from Dyne Pharmaceuticals.
